# A descriptive study on reading instruction provided to students with intellectual disability

**DOI:** 10.1177/17446295211016170

**Published:** 2021-06-16

**Authors:** Rachel Sermier Dessemontet, Anne-Laure Linder, Catherine Martinet, Britt-Marie Martini-Willemin

**Affiliations:** University of Teacher Education of the State of Vaud, Switzerland; University of Teacher Education of the State of Vaud, Switzerland; University of Teacher Education of the State of Vaud, Switzerland; University of Geneva, Switzerland

**Keywords:** developmental disability, intellectual disability, observation, literacy instruction, reading instruction

## Abstract

Little is known about the content and quality of reading instruction provided to students with intellectual disability. This study aimed to describe the reading instruction provided to students with intellectual disability who were not yet readers in self-contained elementary classrooms. The teachers of 24 classrooms participated in the study. Systematic observations of reading lessons, follow-up interviews with the teachers, review of teaching material, and content analysis of students’ individualized education plans were conducted. Findings indicate that phonics and phonological awareness were taught in most of the classrooms. However, phonics was taught systematically, as recommended in the research, in only less than half of the classes. Sight-word instruction and vocabulary instruction were observed in more than half of the classes. Comprehension instruction of texts read aloud by the teacher was observed in only about a third of the classrooms. Recommendations to support teachers in enhancing the quality of reading instruction are provided.

Observational studies play an important role in the field of special education, as they produce evidence that can inform policy and practice (Brantlinger et al., 2005). More specifically, collecting rich descriptive information may allow us to understand to what extent general recommendations or evidence-based practices are actually implemented by professionals in naturalistic contexts. Many studies have investigated the content and quality of reading instruction provided to students with learning disabilities (Walker and Stevens, 2017). In contrast, observational studies on the reading instruction provided to students with intellectual disability are extremely scarce and encompass only a small number of participants (Ahlgrim-Delzell and Rivera, 2015; Ruppar, 2015). Moreover, they were all conducted in the USA. It is urgent to know more about the content and quality of the reading instruction provided to students with intellectual disability in other national contexts, as such information has important implications for planning efficient in-service training for professionals working with students with intellectual disability and for the creation of reading intervention programs. This is especially important, since a significant number of persons with intellectual disability show very poor reading skills (Lemons et al., 2013; Ratz and Lenhard, 2013; Saunders and de Fulio, 2007; Towles-Reeves et al., 2008). This is very worrying because illiteracy is related to limited opportunities for employment and higher chances of poor health in adulthood (Cree et al., 2012; Easton et al., 2010).

Thus, the study presented in this manuscript intends to contribute to a better understanding of the reading instruction provided to elementary school students with intellectual disability in special education classrooms in the French-speaking parts of Switzerland and to assess to what extent the approaches and strategies implemented with these students are research-based.

## Evidence-based reading instruction for typically developing students

Reading instruction encompasses a range of teaching practices aimed at helping students to become independent readers. Learning to read requires the acquisition and integration of a complex set of skills. Students must develop phonemic awareness skills, memorize a large number of letter–sound correspondences, and learn how to use them to decode words (phonics). They must also develop their fluency while reading texts, possess a sufficient vocabulary, and use efficient comprehension strategies to understand what they read (Castles et al., 2018; NICHHD, 2000). These five components of reading instruction outlined by the National Reading Panel (NICHHD, 2000) served as a conceptual framework guiding the data collection in the present study. The research knowledge accumulated on the instruction of these components is briefly summarized hereafter.

### Phonemic awareness

Phonemic awareness refers to the ability to identify and manipulate the sounds of our spoken language. Instruction in phonemic awareness in combination with systematic instruction in letter–sound correspondences is very important to improve the word reading skills of both typically developing children and struggling readers (Castles et al., 2018; NICHHD, 2000). Training syllabic awareness or rime awareness in kindergarten is seen as preparatory activities for the development of phonemic awareness skills, which are more complex (Anthony and Francis, 2005).

### Phonics

Phonics instruction consists in teaching students to identify letter-sound correspondences and how to use them to read and/or spell words (NICHHD, 2000). Meta-analyses have shown that systematic phonics programs were more effective in teaching typically developing children and struggling readers to read words than programs providing unsystematic phonics instruction or no phonics instruction at all (NICHHD, 2000; Torgerson et al., 2006). In a systematic phonics instruction approach, students are taught letter–sound correspondences explicitly in a planned and ordered sequence (NICHHD, 2000). In unsystematic phonics instruction approaches, in contrast, the letter–sound correspondences are taught without a prespecified order, often incidentally in the context of oral or written language activities (NICHHD, 2000).

### Fluency

Efficient word reading skills are necessary but not sufficient to become an expert reader. Students must also learn to read texts fluently. Fluency instruction consists in teaching students to read text with speed, accuracy, and proper expression (NICHHD, 2000). Meta-analyses have shown that fluency can be efficiently improved through repeated reading (Lee and Yoon, 2017; Therrien, 2004).

### Vocabulary

Teaching vocabulary is important to promote comprehension of text, especially for students with reading difficulties (Elleman et al., 2009). Emphasis on richness of contexts in which new words are to be learned, and on providing multiple exposures to new words is recommended (NICHHD, 2000).

### Text comprehension

Text reading comprehension is a complex cognitive process during which meaning is constructed dynamically through interactions between reader and text. Explicit instruction of text comprehension aims at teaching students cognitive strategies or strategic reasoning to help them overcome barriers to comprehension when reading texts (NICHHD, 2000).Meta-analyses showed that text comprehension can be improved through explicit instruction of comprehension strategies (e.g., reciprocal teaching, inference teaching) (NICHHD, 2000; Wright and Cervetti, 2017).

## Research-based approaches and strategies to teach reading to students with Intellectual Disability

For many years, researchers seem to have underestimated the literacy potential of students with intellectual disability. Until recently, learning to decode was often perceived as being too complex for persons with moderate and severe intellectual disability (with IQs lower than 50–55). Systematic reviews show that the reading instruction interventions implemented in studies with these students tended to be restricted mainly to sight-word instruction (Browder et al., 2006; Roberts et al., 2013). Students were taught to recognize words visually, without treating the letter–sound correspondences that compose them.

However, during the last two decades, there has been an increase in studies investigating the effects of interventions aiming at teaching phonological awareness skills and phonics to students with intellectual disability, as observed in the systematic review from Hill (2016). The findings from a recent meta-analysis showed that systematic phonics instruction adopting a direct instruction approach is an evidence-based practice to teach students with intellectual disability to decode (Sermier Dessemontet et al., 2019). More importantly, the findings of several studies conducted recently indicate that students with moderate and severe intellectual disability can progress in their phonological awareness skills and their letter–sound knowledge, and can acquire decoding skills if they benefit from intensive and systematic reading instruction applying research-based techniques (Ahlgrim-Delzell et al., 2014, 2016; Allor et al., 2014; Bradford et al., 2006; Browder et al., 2012; Flores et al., 2004; Lemons et al., 2012, 2015; Tucker Cohen et al., 2008).

Studies conducted on reading comprehension instruction among students with intellectual disability remain scarce, but suggest that explicit instruction in comprehension strategies, for example reciprocal teaching or inference teaching, is a promising intervention for these students (Alfassi et al., 2009; Lundberg and Reichenberg, 2013; Van den Bos et al., 2007). Furthermore, it is possible to foster both literal and inferential comprehension of texts among students with intellectual disability who are not yet able to read, through shared story reading (Browder et al., 2011; Hudson and Browder, 2014; Hudson and Test, 2011; Hudson et al., 2014; Mims et al., 2012). Shared story reading consists in reading a text aloud to students in an interactive reading style, for example encouraging discussion about events and vocabulary within the text and asking questions promoting students’ literal and inferential comprehension of the text during reading (Hudson and Test, 2011).

## The content and quality of the reading instruction provided to students with Intellecual Disability

The few observational studies conducted on reading instruction provided to students with intellectual disability have occurred in special education classrooms for students with moderate and severe intellectual disability in the USA. Ahlgrim-Delzell and Rivera (2015) observed the literacy instruction provided by 12 teachers (6 in 2004 and 6 in 2010) in US special education classrooms (K–5th grade). In 2004, all teachers tended to focus on vocabulary instruction (sight-word instruction, picture identification, or matching a picture to another picture or a word representing the picture). Many of them (67%) also taught concepts of print (e.g., understanding that texts or words are read from left to right) and comprehension. Comprehension activities targeted literal comprehension of the texts read aloud by the teachers. Some teachers (33%) also taught alphabet knowledge (letters’ names). None of them included phonological awareness or phonics instruction in their lessons. Nevertheless, the researchers observed that the quality of reading instruction improved after the teachers participated in a literacy study they conducted that involved 1 day of in-service training, as well as coaching from the research team. In 2010 (1 year after the researchers left the classrooms), half of the teachers trained students in phonological awareness and two-thirds of them taught phonics during reading lessons. All the teachers taught comprehension, half of the time fostering inferential comprehension of texts. A greater use of explicit and systematic instruction was also observed.

In another study, Ruppar (2015) investigated the reading instruction provided by four teachers to eight students with moderate or severe intellectual disability. Her findings indicate that the reading activities provided to these students focused most often on vocabulary instruction (67%). Comprehension instruction was also observed during half of the activities (51%), done through questioning students about their literal comprehension of texts read aloud by the teacher. Phonics and phonemic awareness were observed much less often (8% and 3%). Many teachers seem to have low expectations toward these pupils, which impacts their curricular choices and limits the instructional opportunities they provide to their students (Ruppar, 2017; Ruppar et al., 2015).

As a whole, these results suggest that many students with moderate and severe intellectual disability in special education classrooms may not have access to comprehensive reading instruction. However, current teaching practices must be investigated in other national contexts and in a larger number of classes in order to be better understood, so that levers of action to improve access to comprehensive reading instruction for students with intellectual disability can be found.

## The present study

As highlighted, studies on the reading instruction provided to students with intellectual disability are clearly lacking. A better knowledge of the current teaching practices is extremely important to identify meaningful recommendations to help teachers of these students enhance the quality of reading instruction. The purpose of this observational descriptive study was to describe the reading instruction provided in elementary special education classrooms for students with intellectual disability. Descriptive studies may allow us to understand to what extent evidence-based practices are actually implemented by professionals in naturalistic contexts (Brantlinger et al., 2005). In this study we focused on special education classrooms for students with intellectual disability, since in many European and North-American countries, the majority of students with moderate and severe intellectual disability are schooled in this setting despite recommendations and policies in favor of inclusive education (Flatman Watson, 2009; Göransson et al., 2011; Jahnukainen, 2015; Kleinert et al., 2015; Kurth et al., 2014; Miškolci, 2016; Pijl, 2016; Sermier Dessemontet et al., 2012; Starczewska et al., 2012).

More specifically, the following research questions were investigated: What components of reading instruction (phonological awareness, phonics, vocabulary, comprehension, fluency, sight-word recognition) are taught during reading lessons conducted with elementary students with intellectual disability who are not yet readers? How are these components taught? What components of reading are targeted as learning goals in the individualized education plans (IEPs) of these students?

## Method

### Participants

Teachers in special education classrooms for 6- to 12-year-old students with intellectual disability were recruited in four different French-speaking regions of Switzerland. In these regions, many students with mild intellectual disability are included in general education classrooms during kindergarten and the first years of elementary school (Sermier Dessemontet et al., 2012). Thus, students in self-contained special education classrooms for students with intellectual disability usually tend to have more severe limitations of intellectual functioning and/or adaptive behavior (Sermier Dessemontet et al., 2012). However, these classrooms often comprise a heterogeneous group of students with mild, moderate, and severe intellectual disability.

The teachers of 24 special education classrooms for elementary school students with intellectual disability participated in the study. Teachers signed a written consent form. The classrooms each included three to eight students with intellectual disability (*M* = 5.13, *SD* = 1.33). Half of the classrooms included between one and five students with autism spectrum disorder and intellectual disability. In 16 classrooms, all the students were described by their teachers as not yet being able to decode simple syllables (e.g. ra, si, lu, mo) or words (*n* = 83). These students are described as non-decoders in Table 1. In the eight other classrooms, most students (*n* = 21) were not yet able to decode simple syllables or words, while a few (*n* = 7) were either beginning to decode syllables and words (described as beginning decoders in Table 1), or were able to read short connected texts autonomously (*n* = 12) (described as readers in Table 1).

**Table 1.** Description of the classes.

Class	Teacher 1		Teacher 2		Students		
	Training	Experience	Training	Experience	N	Age	Reading skills
1	T	8	—		5	9–14	non-decoders
2	T	27	T	6	3	8–10	non-decoders
3	T	6	—	—	6	4–8	non-decoders
4	T	12	T	12	6	6–10	non-decoders
5	U	4	U	0	4	5–8	non-decoders
6	T	25	—	—	5	10–12	2 non-decoders, 3 readers
7	T	15	BT	1	7	7–9	non-decoders
8	T	5	T	13	6	8–15	4 non-decoders, 2 readers
9	BT	3	—	—	5	7–13	non-decoders
10	U	15	—	—	3	7–11	2 non-decoders, 1 reader
11	U	27	—	—	4	7–9	2 non-decoders, 1 beginning decoder, 1 reader
12	T	1	—	—	3	6–10	1 non-decoder, 2 readers
13	U	20	U	6	5	6–12	non-decoders
14	T	2	—	—	4	8–10	non-decoders
15	U	15	T	13	7	7–8	non-decoders
16	BT	5	U	7	6	9–11	non-decoders
17	T	2	—	—	5	8–10	non-decoders
18	T	3	—	—	6	9–11	2 non-decoders, 1 beginning decoder, 3 readers
19	T	3	BT	4	8	9–11	4 non-decoders, 4 beginning decoders
20	T	10	BT	10	5	8–10	non-decoders
21	T	15	BT	5	7	7–9	non-decoders
22	U	13	—	—	5	7–10	4 non-decoders, 1 beginning decoder
23	U	5	—	—	4	8–10	non-decoders
24	T	13	—	—	4	6–8	non-decoders

*Note*: Experience = years working as a SET; T = trained special education teacher; BT = being trained as a special education teacher; U = not trained as a special education teacher, SET = special education teacher.

In 17 classrooms, one teacher taught alone most of the days of the week (one teacher working full-time or two teachers working both half-time) seconded in the majority of cases by an aide. In seven classrooms, two teachers taught together 1–4 days of the week, often seconded with an aide present between half-time and full-time. In the French-speaking parts of Switzerland, aides are usually young persons who want to begin a bachelor’s degree program to become social educators but are required to work 1 year before being admitted.

More than half of the professionals working as special education teachers were trained in special education (54%) (master’s degree), while some of them (17%) were completing a master’s degree program to become special education teachers at the time of the study. Most of them had had previous training as social educators. The other professionals had no training in special education (29%); most of them were social educators. In Switzerland, social educators provide support to people with disabilities or social and/or professional difficulties, in various settings (homes, institutions, protected apartments, workshops, vocational or social insertion centers, education centers, etc.). Their training focuses on the acquisition of knowledge in social work, sociology, anthropology, psychology, politics in law, economics, and ethics (University of Applied Sciences and Arts of Western Switzerland, 2019). Information about teachers and students are presented in Table 1.

### Data collection method

Several data collection methods were used: (1) systematic observations of reading lessons and review of teaching material, (2) short individual follow-up interviews with the teachers, and (3) a content analysis of students’ IEP. Data collection was conducted 2 months after the beginning of the school year and lasted 2 months (November–December). Observations were conducted by two researchers who spent a total of four school periods in each classroom. The teachers were instructed to conduct one lesson of reading instruction for their students who were not yet able to read during each observation session. They were told to make the lesson as similar as possible to what they usually did. The researchers used an adapted version of literacy subscale of the Early Language and Literacy Classroom Observation (ELLCO) (K–6), research edition (Smith et al., 2008). This observational tool is aligned with the conceptual framework on reading instruction outlined by the National Reading Panel (NICHHD, 2000). It allows for a close inspection of the five components of reading instruction described in the introduction. Moreover the literacy subscale of the ELLCO has a good inter-rater reliability (88%) and internal consistency (Cronbach’s α = .73–.84) (Smith et al., 2008). In the original version of the ELLCO, the observer must rate teaching practices in each component as *exemplary* (4), *strong* (3), *basic* (2), *inadequate* (1), or *deficient* (0), based on descriptive indicators. In this study, we were interested in describing teachers’ reading instruction practices. Therefore, instead of quantitatively rating the quality of teaching, we used the dimensions of the ELLCO and their indicators as open categories to systematically collect rich descriptive data on each of them. Moreover, observers were instructed to take pictures of the teaching materials used. The two observers received initial training in using the adapted version of the ELLCO, one training session (3 hours’ observation in two classrooms that participated in another study conducted by the research team), and a re-training session. Debriefings were conducted regularly during the 2 months of observations.

The short follow-up interview included in the ELLCO, completed with a few questions, was conducted with the teachers after all the observations had taken place. This interview was used, as recommended in the ELLCO manual, “to gather information to supplement or provide context for observed data” (Smith et al., 2008: 23). This semi-structured interview lasted between 15 and 20 minutes. It included questions such as: “What are the current curriculum that you are working on in your classroom these days?,” “Do you use any particular methods or programs for helping students become readers?,” “How do you use books in your class?”

Additionally, two IEPs of students with intellectual disability who were not yet decoders were collected from each classroom, except for one class in which the teacher did not provide the students’ IEPs despite repeated requests. When several students in the classroom corresponded to our criteria (being 6- to 12-year old, and non-decoder), two IEP were chosen randomly. The purpose of collecting IEP’s was to assess on the basis of another data source than the observations, which components of reading instruction were taught, or at least planned to be taught, to students with intellectual disability that were not yet decoders (methodological triangulation).

### Data analysis

**Figure 1.**
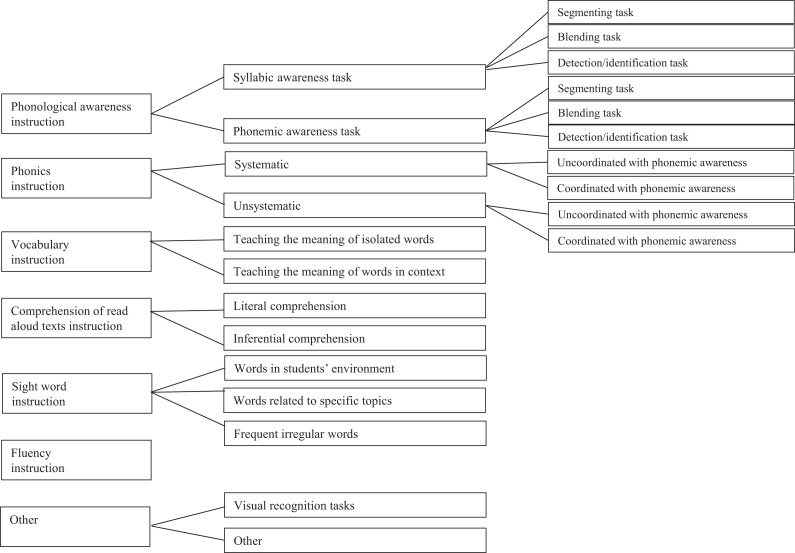
Analysis tree.

The observations (descriptive notes on the reading lessons and photographs of the teaching materials) were coded using a coding guide. In a first step, an analysis was conducted using a-priori main categories: phonological awareness, phonics, vocabulary, comprehension of texts read aloud by the teacher, and fluency instruction as defined by the National Reading Panel (NICHHD, 2000). Sight-word instruction was also used as an a-priori category, since it seems to be often used with students with moderate or severe intellectual disability (Ahlgrim-Delzell and Rivera; 2015; Ruppar, 2015). Sight-word instruction was defined as teaching students to recognize words visually without treating the letter-sound correspondences that compose them. Two researchers coded all the data in these a-priori categories independently. Observed teaching practices that did not fall into our a-priori categories were coded in an “Other” category. A high inter-rater agreement percentage was obtained (96%). Discrepancies were resolved by consensus reached in meetings to discuss the coding. In a second step, a more in-depth analysis was conducted using categories identified after reviewing the data coded in each main category several times (e.g. systematic vs. unsystematic instruction in the category phonics). A high inter-rater agreement percentage was obtained when coding data in these subcategories (94%). A coding tree is presented in Figure 1. The codes for each category and subcategory were entered in an Excel file with one row devoted to each class. The raw data collected during the short teacher follow-up interview was extracted and entered in this Excel file for each question. As recommended in the ELLCO, this data was used to provide context for interpreting the observed data.

The learning goals related to oral and written language were extracted from the students’ IEPs. They were coded with a coding guide, with a-priori categories: phonological awareness, phonics (letter–sound knowledge/decoding skills/spelling skills), vocabulary, fluency, comprehension of texts read aloud by the teacher, and sight-word recognition. Two researchers coded independently a third of the collected data, selected randomly. A high inter-rater agreement percentage was obtained (95%). Discrepancies were resolved by consensus reached in meetings to discuss the coding. The codes for each category were entered in the previously mentioned Excel file

In order to establish the credibility and trustworthiness of the findings several steps were undertaken. First, we used data triangulation, combining observations with IEP’s analysis (data and methodological triangulation). Second, the analysis of the data (e.g. construction of the coding guide) and interpretation of the data were regularly discussed between the four co-authors, each one having complementary expertise in the field of reading instruction for struggling students and/or students with intellectual disability (investigator triangulation). Third, an external auditor (a researcher from another country having conducted studies on reading instruction with students with intellectual disability) reviewed the coding guide, as well as the findings’ interpretation and provided critical feedback on both. For example, she recommended a better distinction of different types of sight-word instruction practices in the coding guide and provided useful inputs for the refinement of subcategories for comprehension instruction.

## Results

The findings will be presented for each of the following categories, in relation with the five components of reading instruction outlined by the National Reading Panel (NICHHD, 2000): phonological awareness, phonics, vocabulary, fluency, comprehension, and sight-word instruction. Table 2 presents a synthetic vision the presence of these six components of reading instruction during the observed reading lessons.

**Table 2.** Percentage of classes providing instruction in each category during the observed lessons.

Instruction	Percentage of classes
Phonological awareness	75%
Phonics	92%
systematically	46%
unsystematically	46%
Vocabulary	54%
Comprehension	38%
Fluency	0%
Sight-word	58%

### Phonological awareness

Phonological awareness instruction was observed in 75% of the classrooms (18 classes). It was provided most often with all the students present in the class (47%) or in a small-group format (two to three students) (21%). In six classes, students were trained in syllabic awareness skills, mainly segmenting words in syllables and syllable identification (e.g., identifying a word containing a given syllable or matching words with the same syllable). Students were trained in blending syllables to form words in two classrooms. Phonemic awareness skills were trained in 18 classes, mainly phoneme identification (e.g., identifying words beginning with a given phoneme or containing a given phoneme). Explicit exercises to train students to blend phonemes into syllables or words orally—a core phonemic skill for reading words—were observed in only one class. However, teaching students to blend phonemes was also observed in five other classes, embedded in a syllable-decoding activity. Segmentation of words in phonemes was also trained in two classes, embedded in a syllable- or word-spelling activity.

The teachers often used games or activities involving sorting images. Teachers reported in the follow-up interviews that these activities were inspired by published phonological awareness programs for typically developing children (Dorner, 2013; Goigoux et al., 2004; Monney et al., 2016). A few teachers reported using worksheets downloaded on the Internet. In addition, in only about half of the classrooms (48%) did students’ IEP include learning goals related to phonological awareness (e.g. “segment words into syllables,” “identify words beginning with a given sound” or “locate a phoneme into a word (at the beginning, in the middle or at the end of the word).”

### Phonics

Phonics instruction was observed in the majority of classrooms (92%). It was provided either in one-to-one format (36%), small-group format (two to three students) (24%), together with all students present in the classroom (four to eight students) (27%), or in independent seatwork format (13%). However, letter–sound correspondences were taught systematically (as is recommended for typically developing children, struggling readers, and students with intellectual disability) only in less than half of the classrooms (46%). Teaching phonics systematically is defined as teaching letter–sound correspondences explicitly in a planned and ordered sequence from less to more complex. The lessons provided by the teachers using a systematic approach focused on teaching a new and unknown letter–sound correspondence or reviewing three to four letter–sound correspondences that had been previously taught. These lessons included phonemic awareness instruction related to the targeted letter–sound correspondences, as recommended by the research.

In contrast, in approximately half of the classrooms, teachers used an unsystematic approach to teach phonics (46%). In these classrooms some teachers exposed students to several new letter–sound correspondences (>8) at the same time during authentic language activities (e.g., in relation to a text read aloud to the students, to a special event such as a visit to a zoo, or to a literacy project such as creating a book). Other teachers, exposed students to several new letter–sound correspondences (>8) at the same time during lessons with very disparate and disorganized content (e.g., a lesson included asking a student to recognize globally a list of syllables, a phonemic awareness activity (identification of words containing the phoneme /y/), a syllabic awareness activity (identifying two words beginning and then ending with the same syllable—syllables completely different from the syllables identified in the first activity), and a spelling activity (dictation of words apparently chosen at random with many letter-sound correspondence not known by the student).

Direct instruction in strategy to decode syllables or words using modeling was also never observed. However, several teachers moved two moveable letters or two letter cards toward each other to help their students understand blending while decoding syllables. In the majority of the classrooms (87%), students’ IEPs included learning goals targeting letter–sound knowledge, and in half of the cases decoding skills and spelling skills as well (e.g. “learn the sound of several letters,” “decode syllables and short words,” “write syllables and short words”).

### Vocabulary

Vocabulary instruction was observed in approximately half of the classrooms (54%) during the observed reading lessons. The majority of vocabulary instruction took place with all the students in the classroom (four to eight students) (69%). Most frequently, students were taught the meaning of isolated words, often with the help of pictures. In six classes, vocabulary instruction was embedded in a read-aloud activity (stories, song or poetry read aloud by the teacher) allowing students to understand the meaning of new words in a given context. Learning goals related to vocabulary were found in the IEPs of the students in 61% of the classrooms (e.g. “enrich lexicon on different topics (clothing, cooking, classroom environment)” or “name new words”).

### Comprehension

Comprehension instruction for texts read aloud by the teachers was observed in only 38% of the classrooms. It was often provided to all the students in the classroom, in whole-group format (four to eight students) (55%) or small-group format (two to three students) (27%). These teachers used shared story reading to foster their students’ literal comprehension of the text, clarifying the meaning of unknown words for the students, questioning them about characters or events, or asking them to summarize the story or a part of the story in their own words, sometimes with support from pictures in the book. Five teachers (21%) also asked their students inferential questions fostering deeper comprehension of the text (e.g., asking them to make predictions about events and inferences about the emotions or thoughts of the principal characters of a story). Shared story reading focused exclusively on narrative texts during the observed lessons.

In the follow-up interviews, many teachers (79%) reported reading books aloud to their students once a week or once every 2 weeks. However, only a third of them reported exploiting those read-aloud stories to teach comprehension and/or vocabulary (38%), which is consistent with the observations. The other teachers talked about “gift-reading” or “pleasure-reading” (*lecture-cadeau* and *lecture-plaisir* in French), which refers to the teacher reading books aloud to their students without interacting with them around its content. Learning goals related to understanding texts read aloud by the teacher were seldom found in the students’ IEPs (43%) (e.g. “order chronologically the events of a story using pictures,” “retell a story in his own words” or “identify main characters of a story”). This tends to confirm that this component of reading instruction is under-emphasized in the reading instruction provided to students with intellectual disability who are not yet able to read.

### Fluency

No instructional fluency instruction at the text level was observed during reading lessons, which is coherent with the fact that only lessons for students that were unable to read were observed in the present study. Indeed, fluency instruction is recommended for students that are able to decode autonomously (NICHHD, 2000).

### Sight-word recognition

Sight-word instruction was observed in 58% of the classrooms. Most of the time it was provided in small-group format (43%) or with all the students present in the classroom (21%). In the majority of the classrooms, sight-word instruction was restricted only to a small number of words, mainly the students’ names, the teachers’/professionals’ names, and/or information appearing in the students’ daily program (e.g., the name of the day, activities engaged in). In four classrooms, sight-word instruction was used to teach students to visually recognize a larger number or wider range of words (e.g., words encountered in a book read aloud to the students, in the school’s meal menu, or in a child’s favorite cartoon). The use of sight-word instruction to teach students to recognize frequent irregular words was also observed in one classroom. Learning goals related to sight-word recognition were found in the IEPs of the students in approximately half of the classrooms (52%) (e.g., “recognize familiar words such as days of the week or seasons” or “recognize one’s name and the names of classmates”).

### Other practices

Some observed practices did not fall into our a-priori categories. In a few classes, especially in the two classes where phonics was not taught, students were provided with visual recognition tasks during reading lessons: pairing identical forms or pictures of objects (13%), pairing identical letters (21%), or pairing identical words (8%). During these tasks, the teachers sometimes named some of the letters’ name, but never the sounds made by the letters, and never asked the students to do it. Such activities can therefore not be considered as teaching phonics. These activities were described by the teachers as training “pre-requisites to reading” in the follow-up interviews. Other activities were observed only in one of the classes (4%): revising the order of the letters in the alphabet, identifying the titles in several book covers, and a story read-aloud by the teacher without interactions with the students around its content (described as “gift-reading” by the teacher in the follow-up interview).

## Discussion

The goal of this study was to describe the reading instruction provided in elementary special education classrooms for students with mild, moderate, and severe intellectual disability who were not yet readers. As a whole, our findings suggest a significant research-to-practice gap in the way reading instruction is provided in many special education classrooms for students with intellectual disability. The extent of this gap varied depending on the class and the reading component analyzed. Our findings suggest that in the majority of the classrooms, teachers tried to teach phonics and phonological awareness to these students and included learning goals related to phonics in the students’ IEPs. However, only slightly less than half of them (46%) taught phonics with a systematic approach and in combination with phonemic awareness, as recommended for typically developing children, struggling readers, and children with intellectual disability. Approximately half of the teachers used an unsystematic approach to teach phonics (46%). This type of unsystematic approach, exposing students to several unknown letter–sound correspondences at the same time in each lesson, is probably not efficient enough to allow students with intellectual disability to learn all the correspondences needed to be able to read. Students with intellectual disability, especially moderate intellectual disability, are known to need more intensive and regular practice to memorize letter–sound correspondences and learn how to use them to decode (Allor et al., 2014; Sermier Dessemontet et al., 2019). Even among typically developing children, unsystematic phonics instruction is less efficient than systematic phonics instruction (Castles et al., 2018; NICHHD, 2000).

Our findings also suggest that the other components of reading were neglected in many classrooms. Vocabulary was taught during reading lessons and included in the students’ IEPs in approximately half of the classrooms. Listening comprehension instruction through shared story reading was observed in only about a third of the classrooms and also seldom found in the students’ IEPs.

### Comparison of our findings with previous studies

On the whole, our findings contrast with those of previous studies, which found that most teachers did not teach phonics or phonological awareness skills in special education classrooms for students with moderate and severe intellectual disability (Ahlgrim-Delzell and Rivera, 2015; Ruppar, 2015). This difference may be due to differences in context, as the classrooms observed in the current study included not only students with moderate and severe intellectual disability but also a few students with mild intellectual disability. Having students with less severe limitations in their classrooms may induce teachers to plan more academic instruction encompassing all their students. Moreover, in Switzerland, recommendations have recently been made to provide students with intellectual disability access to general education curriculum content (CDIP, 2007). Special education teachers working in special education classrooms for students with intellectual disability not only receive incentives to include learning goals from the general education curriculum in their students’ individualized education plans, but also practical help in this process (a written guide with examples of learning goals based on the general education curriculum and/or a canvas for IEPs with a strong link to the general education curriculum).

### Recommendations for supporting teachers to optimize phonological awareness instruction

Phonological awareness instruction was observed in most of the classrooms. It was often provided appropriately with activities inspired by published programs (in French) (Dorner, 2013; Goigoux et al., 2004; Monney et al., 2016). Most teachers would benefit from being taught the technique, which involves stretching continuous sounds (such as /fff/, /mmm/, /sss/) during phonemic awareness activities in order to facilitate their identification or the process of blending them (Allor et al., 2009). Moreover, blending activities and segmenting activities were too seldom observed. Teachers should be taught how to skillfully create and include in their reading lessons short exercises to train students in phoneme blending orally (Allor et al., 2009). Indeed, phoneme blending is a central phonemic awareness skill, the mastery of which is very important when decoding words (NICHHD, 2000). Teachers would also benefit from being instructed in how to teach their students explicitly how to segment words in phonemes before spelling them (Allor et al., 2009). Finally, teachers could be encouraged to include meaningful learning goals related to phonemic awareness in the students’ IEPs.

### Recommendations for supporting teachers to optimize phonics instruction

Teachers that already teach phonics systematically in co-articulation with phonemic awareness would benefit from knowing and integrating in their practices several research-based strategies identified recently to more efficiently teach decoding skills to students with intellectual disability. First, namely, these teachers should be trained to model the targeted skills to their students more often and more effectively, as is recommended in a direct instruction approach (Sermier Dessemontet et al., 2019). They would also benefit from learning to use systems of prompts when providing corrective feedbacks to their students (e.g., constant time delay or least to most intrusive prompts). Systems of prompts have been used successfully in several reading intervention studies conducted among students with intellectual disability (Ahlgrim-Delzell et al., 2014, 2016; Allor et al., 2014; Lemons et al., 2015; Tucker Cohen et al., 2008). Second, these teachers should be trained to teach students decoding skills more explicitly, with a direct instruction approach (Sermier Dessemontet et al., 2019). They could, for example, teach a two-or-three-step decoding strategy: (1) naming the letter–sound correspondences (e.g., “/r/–/a/–/t/”), (2) reading the word while stretching continuous sounds and blending them (e.g., “/rrrraaat/”), and (3) reading the word fast (e.g., “/rat/”) (Allor et al., 2009; Bradford et al., 2006; Flores et al., 2004; Tucker Cohen et al., 2008).

Teachers who do not teach phonics or teach phonics unsystematically would clearly need more thorough in-service training than the aforementioned teachers, including basic knowledge on evidence-based teaching practices in reading and how to apply them concretely for students with intellectual disability. They would benefit from being instructed how to follow a specific and progressive order in the letter–sound correspondences taught. Moreover, they should receive concrete examples of lessons provided with a direct instruction approach and articulating phonemic awareness instruction through phonics.

### Recommendations for supporting teachers to optimize vocabulary and comprehension instruction

Comprehension instruction was not frequently observed in this study. Most of these teachers would benefit from being taught how to teach comprehension and vocabulary through shared story reading to their students, who are not yet able to read short texts by themselves. Shared story reading is an interactive reading experience during which teachers not only read a text aloud, but also support students’ interactions with the reader (the teacher) and the text (Hudson and Test, 2011). Teachers can, for example, ask students to predict story events before reading the story, encourage discussion about events and vocabulary within the story, and ask questions prompting students’ inferential thinking during reading (Allor et al., 2009). Providing teachers with a list of books recommended for students with intellectual disability at different ages, accompanied by short guides on how to question students in order to foster vocabulary enhancement and a literal and inferential comprehension of the text, could be useful to them. For example, Browder et al. (2007) created a literacy lesson plan template that can be applied across changing stories for shared story reading with students with moderate or severe intellectual disability and then taught teachers to use it.

Finding age-appropriate texts that are still accessible for students with intellectual disability becomes more difficult as they grow older. Translating some recommended books in easy-to-read form could help teachers base their lessons on age-appropriate books. Indeed, “easy-read” may facilitate literal comprehension of the text (Sutherland and Isherwood, 2016), allowing teachers to focus their lessons on teaching more complex comprehension strategies. Hudson et al. (2013) also described very useful strategies to adapt grade-level texts for students with moderate or severe intellectual disability and then exploit them for shared story reading.

### Levers of action

Providing professionals working in special education classrooms for students with intellectual disability with in-service reading instruction training seems like a promising lever of action to improve the quality of reading instruction. Our findings suggest that in-service training would be beneficial not only for professionals with no training in special education but also for trained special education teachers, as a research and practice gap was observed in the teaching practices of several trained special education teachers in this study. Viewing students with moderate and severe intellectual disability as having the potential to learn reading skills is a relatively recent development, and teachers’ skills may lag behind. Providing these teachers with knowledge on evidence-based interventions and strategies, as well as success stories of students with moderate and severe intellectual disability who have benefitted from such interventions, could help their expectations evolve and the quality of their instruction improve.

Finally, no published reading intervention program integrating research-based approaches and techniques to teach reading to students with intellectual disability exists in French. Creating a reading intervention program in French, integrating research-based approaches and techniques for students with intellectual disability, may also be an efficient lever of action to optimize reading instruction provided to these students (Ahlgrim-Delzell and Rivera, 2015). Creating teaching material in French for shared story reading also seems to be required.

### Study limitations

Our study enhanced the current knowledge on the content and quality of reading instruction provided to students with intellectual disability in elementary special education classrooms, especially in the Swiss context. Nevertheless, only four school periods were observed in the 24 classrooms participating in our study. Thus, it is difficult to know with certainty if the observations reflect the entire breadth and diversity of the reading practices in the observed classrooms. Furthermore, our findings are not necessarily generalizable in their entirety to other national contexts. Moreover, this study focused solely on instruction provided in self-contained classrooms. Observing reading instruction provided to students with intellectual disability included in general education classrooms may have generated different findings. Finally, more in-depth interviews with teachers would have yielded useful insights about teachers’ instructional choices and practices. Additional studies should be conducted in other countries to acquire a broader image of the reading instruction provided to students with intellectual disability and the impact of individual and contextual variables influencing these practices.

## Conclusion

Our study offered a description of the reading instruction provided to students with intellectual disability in elementary special education classrooms in the French regions of Switzerland. It highlighted the importance of having a better knowledge of the teachers’ current teaching practices in order to plan efficient in-service trainings. Once the present study was completed, the participants were provided with an in-service training tailored to their needs. Moreover, this study’s findings gave momentum to the creation of teaching material in French to help bridge the research-to practice gap (Sermier Dessemontet et al., 2021).
